# Mitochondrial targeted HSP90 inhibitor Gamitrinib-TPP (G-TPP) induces PINK1/Parkin-dependent mitophagy

**DOI:** 10.18632/oncotarget.22287

**Published:** 2017-11-06

**Authors:** Fabienne C. Fiesel, Elle D. James, Roman Hudec, Wolfdieter Springer

**Affiliations:** ^1^ Department of Neuroscience, Mayo Clinic, Jacksonville, FL 32224, USA; ^2^ Mayo Clinic Graduate School of Biomedical Sciences, Jacksonville, FL 32224, USA

**Keywords:** PINK1, parkin, mitophagy, mitochondrial UPR, Gamitrinib

## Abstract

Loss-of-function mutations in *PINK1* or *PARKIN* are associated with early-onset Parkinson’s disease. Upon mitochondrial stress, PINK1 and Parkin together mediate a response that protects cells from the accumulation of harmful, damaged mitochondria. PINK1, the upstream kinase accumulates on the mitochondrial surface and recruits the E3 ubiquitin ligase Parkin on site to ubiquitylate substrate proteins. The joint activity of both to generate phosphorylated poly-ubiquitin chains on the mitochondrial surface induces the recruitment of autophagy receptors and eventually whole organelles are cleared by autophagy. While this pathway is generally accepted to occur upon chemical uncoupling of mitochondria, the (patho-) physiologic relevance has been questioned. However, few studies have indicated that PINK1 and Parkin are also activated upon accumulation of misfolded proteins in the mitochondrial lumen upon overexpression of ΔOTC (Ornithine transcarbamylase). Here, we used the mitochondrial targeted HSP90 inhibitor Gamitrinib-triphenylphosphonium (G-TPP), an anti-cancer agent, to chemically interfere with mitochondrial protein folding. G-TPP treatment induced PINK1 accumulation, ubiquitin phosphorylation at Ser65, Parkin activation and its recruitment to mitochondria was specific for mitochondrial HSP90 inhibition and largely independent of mitochondrial membrane depolarization. Mitophagy induction was observed by monitoring autophagy receptor recruitment and the mitoKeima reporter. Importantly, mitophagy was not only induced in cancer cells but also in primary human fibroblasts and thereof converted neurons. G-TPP treatment might represent a novel strategy to study PINK1 and Parkin-mediated mitochondrial quality control using a more physiologically relevant stress.

## INTRODUCTION

Parkinson’s disease (PD) is the second most common neurodegenerative disease. Genetic analyses have revealed a number of genes associated with disease [1]. Early-onset forms of PD are frequently caused by mutations in the genes encoding for PINK1 and Parkin [2]. A major breakthrough for the field was that the activities of both, the mitochondrial kinase PINK1 and the cytosolic E3 ubiquitin (Ub) ligase Parkin, could be linked to mitochondrial quality control (mitoQC) [3, 4] and that loss of either gene function disrupts this stress-activated and neuroprotective pathway [4, 5]. In the absence of stress, PINK1 is constitutively imported through the translocase of the outer/inner membrane (TOM/TIM) machinery into mitochondria where it undergoes cleavage by the mitochondrial processing peptidase (MPP) in the matrix and the presenilin-associated rhomboid-like protease (PARL) in the inner mitochondrial membrane (IMM) [6–8]. N-terminally processed PINK1 is transported back to the cytoplasm where it is degraded by the proteasome [9]. Mitochondrial uncoupler such as CCCP and others induce loss of the electrochemical potential across the IMM (ΔΨ) and result in PINK1/Parkin-dependent autophagy of damaged mitochondria (mitophagy).

As a consequence of collapsed mitochondrial protein import, newly synthesized PINK1 inserts into the outer mitochondrial membrane (OMM) where it accumulates and phosphorylates the cytosolic substrate proteins Ub and Parkin at the same conserved residue (Ser65) [10–12]. Auto-inhibited Parkin is activated by PINK1-mediated phosphorylation and by binding to phosphorylated Ub (pS65-Ub) that also acts as the mitochondrial receptor for Parkin [13–15]. Upon activation and recruitment, Parkin ubiquitylates substrate proteins on the mitochondrial surface [2]. The joint enzymatic activities of PINK1 and Parkin increase the abundance of mitochondrial pS65-Ub [16]. While some poly-ubiquitylated proteins are extracted and degraded by the proteasome, autophagy adapters that bind pS65-Ub and the autophagic machinery further facilitate the degradation of the entire organelles in lysosomes [2].

Many mechanistic details of this pathway have been worked out since its discovery. However, several questions still remain unanswered. While robust PINK1 and Parkin activation in cell culture experiments is achieved by using chemicals that promote mitochondrial uncoupling, the (patho-) physiologically relevant stress leading to PINK1/Parkin activation remains uncertain. It has been shown that Parkin protects against dopaminergic cell death in a mouse model that accumulates dysfunctional mitochondria caused by an accelerated generation of mtDNA mutations (Mutator mice) [17]. Few other studies have indicated that PINK1 and Parkin are activated in response to accumulation of misfolded proteins inside the mitochondrial matrix. The expression of a deletion mutant of ornithine carbamoyltransferase (ΔOTC) has been used to prove the accumulation of PINK1 on energetically healthy cells [18].

Here we report the pharmacologic induction of PINK1 and Parkin mediated mitoQC with the mitochondrial targeted chaperone inhibitor Gamitrinib-triphenylphosphonium (G-TPP) [19]. G-TPP targets mitochondrial-localized members of the HSP90 family including TNF receptor-associated protein-1 (TRAP1) and was developed to treat cancer cells where cytosolic HSP90s are abundant inside mitochondria. While G-TPP leads to cell death at high concentrations, low concentrations trigger the induction of mitochondrial unfolded protein response (mitoUPR) [19, 20]. In this study, we have carefully validated the response of cells upon treatment with G-TPP and found bona fide PINK1/Parkin mitoQC induction not only in cancer cells but also in primary human skin fibroblasts and induced neurons. While cytosolic HSP90 appeared to be required for proper folding of the kinase PINK1, mitochondrial HSP90 was needed for protein import and correct assembly of multiprotein complexes as the induction of mitoQC was independent of mitochondrial membrane depolarization, but rather was caused by the accumulation of insoluble proteins inside mitochondria.

## RESULTS

### G-TPP induces PINK1 stabilization and kinase activity

**Figure 1:**
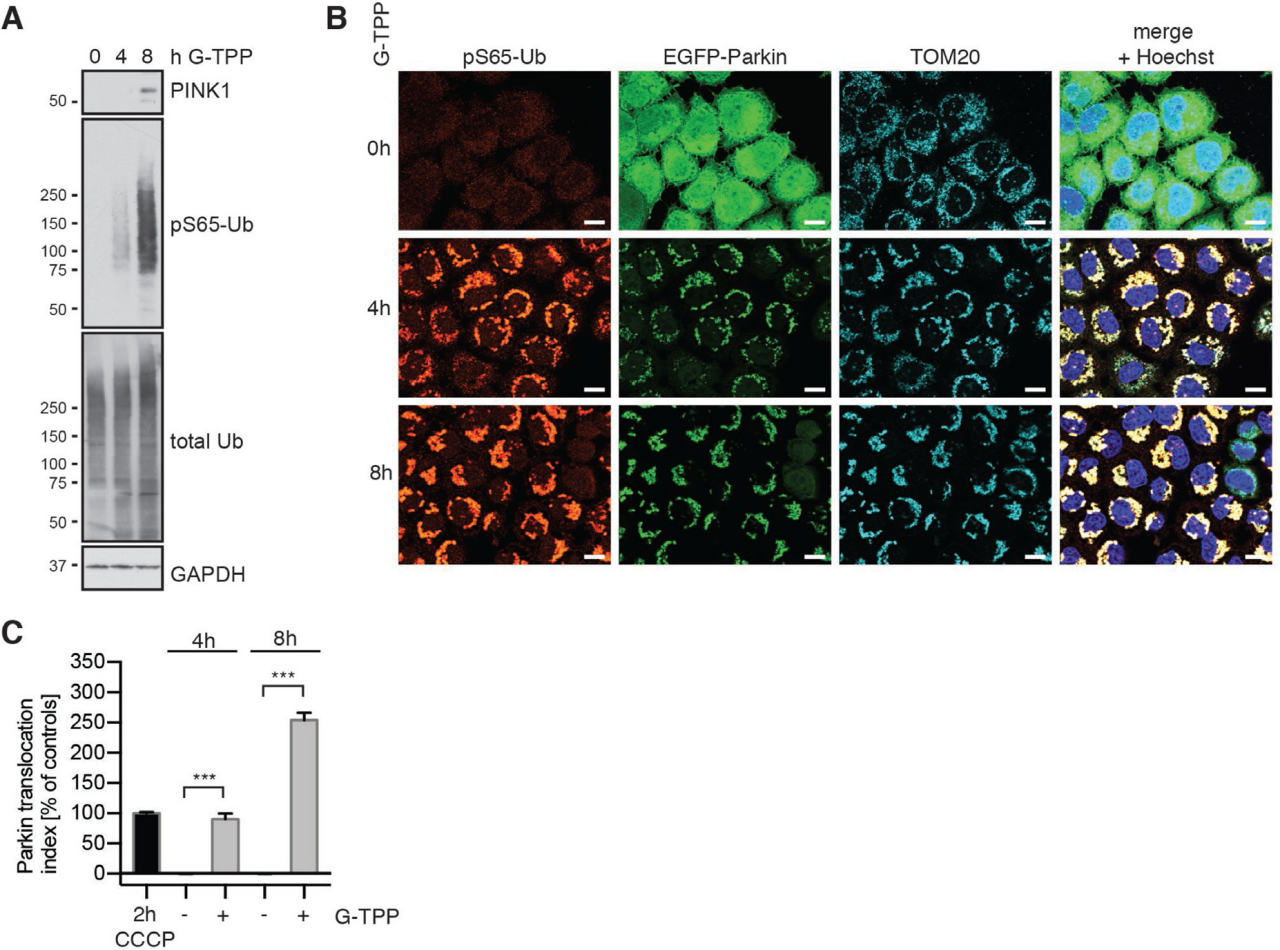
G-TPP induces PINK1 stabilization and kinase activity in HeLa cells (**A**) G-TPP treatment leads to PINK1 stabilization and pS65-Ub induction in HeLa cells. HeLa cells stably expressing untagged Parkin were treated with 10 µM G-TPP for the indicated times. Western blots were prepared with cell lysates and probed with antibodies against PINK1 and pS65-Ub. GAPDH served as a loading control. (**B**) pS65-Ub is induced in G-TPP treated cells and co-localizes with EGFP-Parkin and mitochondria. HeLa cells stably expressing EGFP-Parkin (green) were treated with 10 µM G-TPP for the indicated times and fixed. Cells were stained with antibodies against pS65-Ub (red) and the mitochondrial marker TOM20 (cyan). Scale bars correspond to 10 µM. (**C**) Quantification of Parkin translocation using High Content Imaging. HeLa EGFP-Parkin cells were treated for 4 or 8 h with or without 10 µM G-TPP. CCCP treatment (10 µM for 2 h) was used as a positive control. Cells were fixed, counterstained with Hoechst dye to visualize nuclei, imaged and analyzed using the ratio of cytoplasmic to nuclear EGFP signal [21]. Data was normalized to positive (2 h 10 µM CCCP treatment) and negative (2 h DMSO) controls. G-TPP significantly induced Parkin re-localization to levels similar to or beyond 2 h CCCP treatment. Shown are the mean values of three independent experiments with triplicate wells each ± SEM (one-way ANOVA with Tukey’s posthoc, ^***^p < 0.0005).

In order to determine if mitochondrial TRAP1/HSP90 inhibition activates mitophagy, we first tested the ability of G-TPP to induce PINK1 stabilization and kinase activity. HeLa cells stably expressing untagged Parkin were treated for different time points and accumulation of PINK1 and Ub kinase activity were analyzed by western blot. As expected PINK1 protein was undetectable in untreated cells, but accumulated 8 h after treatment with G-TPP along with the increase of pS65-Ub signal (Figure 1A). Given the higher sensitivity of the pS65-Ub antibody and/or the abundance of epitopes, phosphorylation of pS65-Ub in HeLa cells was detected even before PINK1 protein. In line with western blot analyses, immunofluorescence staining of HeLa cells stably expressing EGFP-Parkin showed a robust induction of pS65-Ub that was co-localized with mitochondria (Figure 1B). In addition, we observed translocation of EGFP-tagged Parkin from the cytosol to mitochondria and clustering of mitochondria upon G-TPP treatment. In order to quantify the effects of G-TPP on Parkin translocation, we used a well-established high content imaging (HCI) assay [21, 22]. HeLa cells stably expressing EGFP-Parkin were seeded in optical plates, treated with 10 µM G-TPP for 4 h or 8 h and analyzed (Figure 1C). G-TPP treatment robustly induced Parkin translocation over time, though with slower response compared to a CCCP control treatment (2 h, 10 µM).

### G-TPP induces Parkin E3 Ub ligase activity

**Figure 2:**
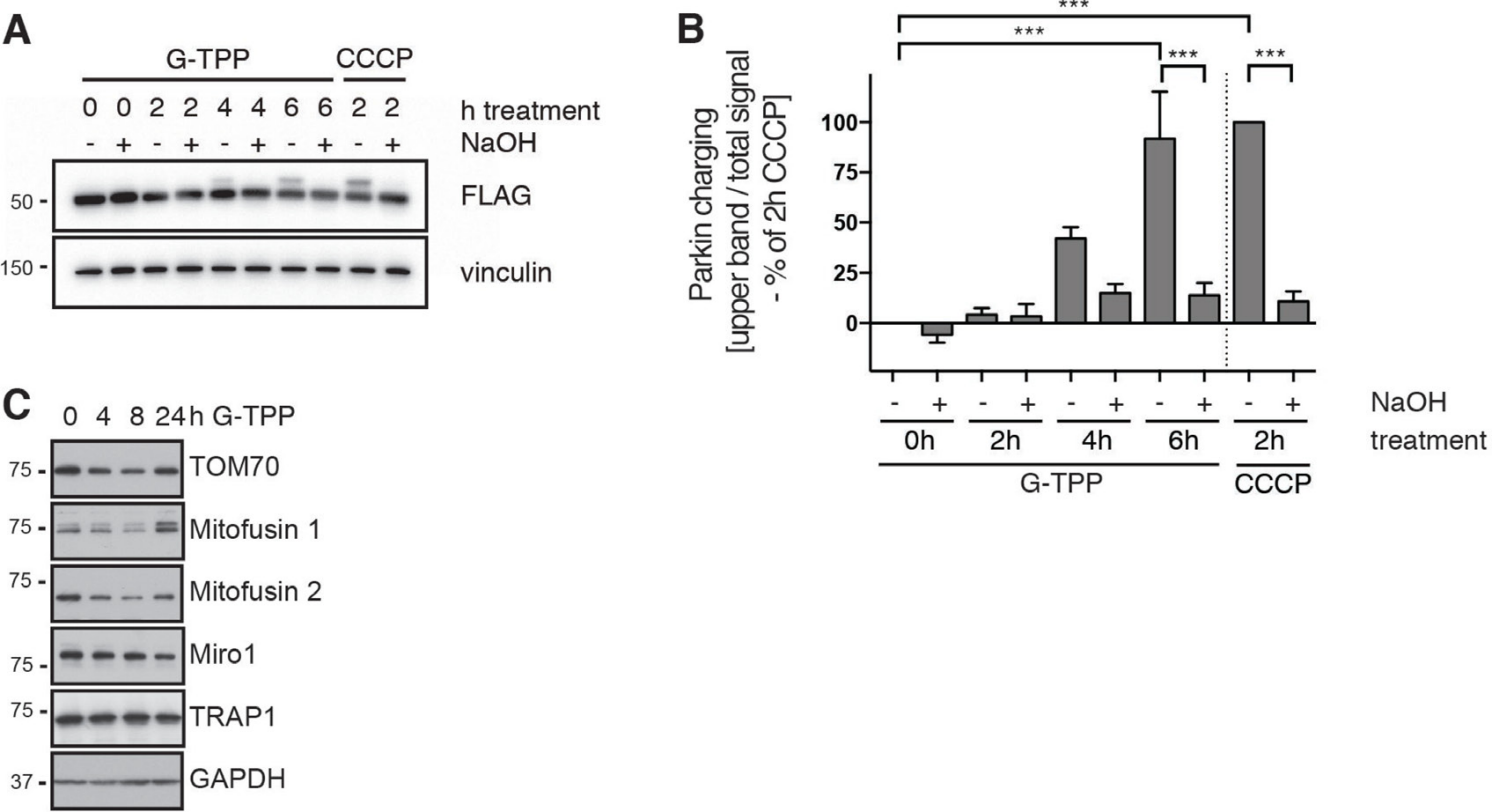
G-TPP induces Parkin Ub-charging and substrate degradation (**A**, **B**) G-TPP leads to Ub-charging of the active site mutant Parkin C431S. HeLa cells stably expressing 3xFLAG-Parkin C431S were treated with 10 µM G-TPP for the indicated times. As positive control some cells were treated with 10 µM CCCP for 2 h. (A) Western blots were prepared with cell lysates and probed with antibodies against FLAG. Ub-charged Parkin can be observed as 8 kDa band shift that collapses again with NaOH treatment. (B) Densitometric quantification of Parkin Ub-charging normalized to 2 h CCCP treatment. Shown is the average of three independent experiments ± SEM (one-way ANOVA with Tukey’s post-hoc test, ^***^p < 0.0005). (**C**) HeLa cells stably expressing untagged Parkin were treated with G-TPP for the indicated times. Western blots were prepared with cell lysates and probed with antibodies against Parkin substrates (Mitofusin 1 and 2, TOM70 and Miro1). Levels of Parkin substrates initially decreased but then recovered during the time course. Levels of TRAP1 were unchanged upon G-TPP treatment.

To directly assess Parkin E3 Ub ligase function, we first used a biochemical readout employing an active site mutant of Parkin [16, 21]. Upon activation, Parkin’s catalytic center Cys431 is charged with an Ub moiety in a thioester bond before transfer onto a lysine residue of a substrate protein. While this complex is very transient, a C431S substitution allows trapping of Ub through formation of a more stable oxyester bond. Ub charging of Parkin C431S results in an 8 kDa band shift and is chemically cleavable by NaOH. HeLa cells stably expressing 3xFLAG-Parkin C431S were treated with 10 µM G-TPP for different times and lysates were analyzed by western blot. While we did not find Ub charging of Parkin after only 2 h of G-TPP, 4 and 6 h treatment resulted in increased levels of Ub-bound Parkin (Figure 2A). Consistent with an initial slower PINK1 accumulation, pS65-Ub increase, and Parkin translocation upon G-TPP treatment compared with CCCP (2 h, 10 µM), longer times of mitochondrial HSP90 inhibition robustly induced Ub-charging of Parkin (Figure 2B).

We next used HeLa cells expressing untagged Parkin and a panel of antibodies directed against known OMM Parkin substrates to monitor their degradation. G-TPP treatment induced reduction of TOM70, Mitofusins (1 and 2) and Miro1 levels over time. This decrease seemed of a more transient nature as levels of some substrates recovered after 24 h of treatment with G-TPP (Figure 2C). This was also consistent with our observations in immunofluorescence experiments, where 24 h after G-TPP a portion of the cells looked like untreated cells (data not shown). However, potential compensatory changes on TRAP1 protein levels were not observed.

### G-TPP induces mitophagy

**Figure 3:**
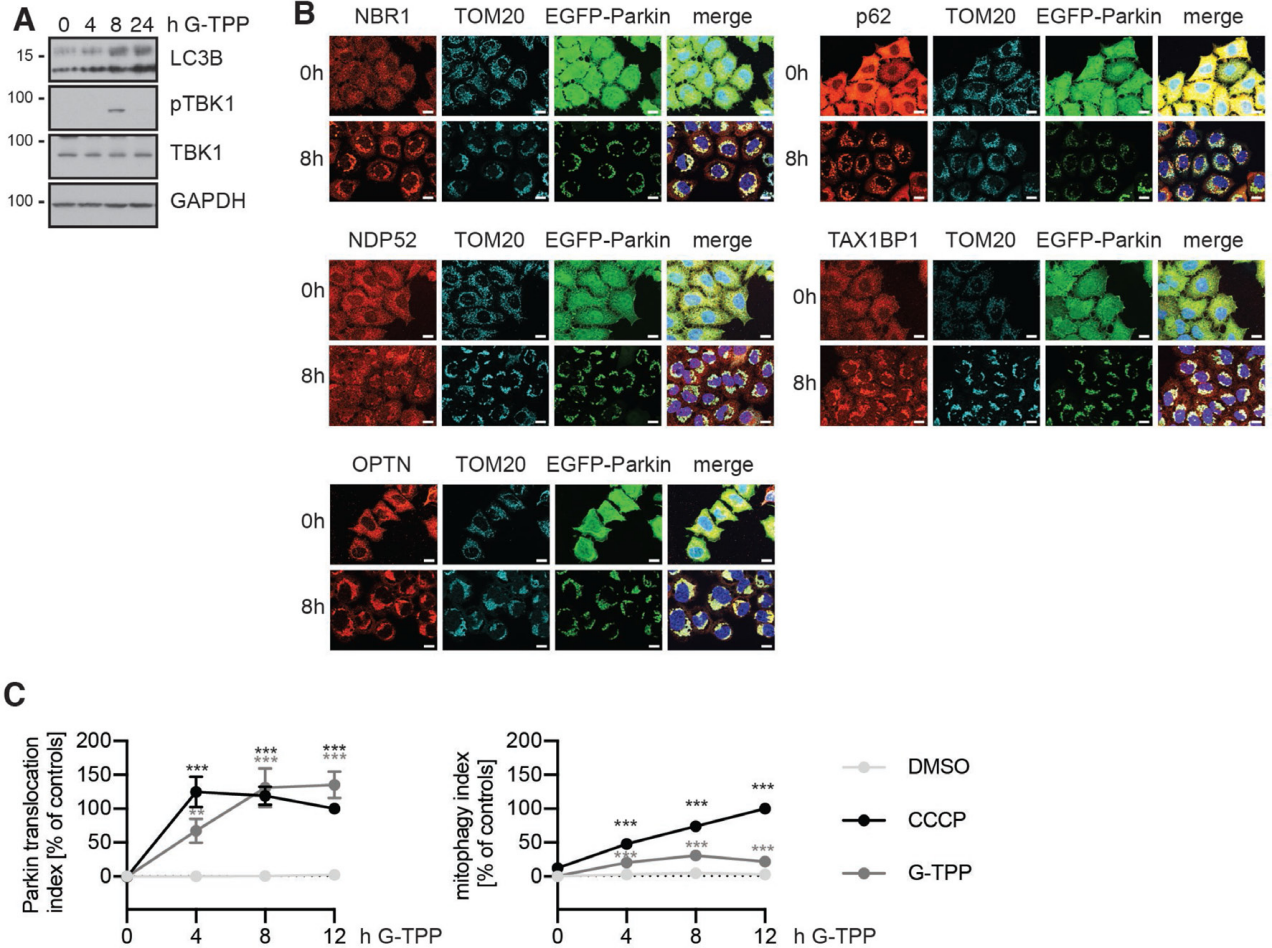
G-TPP leads to recruitment of autophagy adapters and degradation of mitochondria (**A**) HeLa cells stably expressing untagged Parkin were treated with 10 µM G-TPP for 8 h. Western blots were prepared from cell lysates and probed with antibodies against LC3, phospho-TBK1 (Ser172) and TBK1. GAPDH was used as a loading control. Upon 8 h the levels of LC3-I and LC3-II were both increased. At 8 h after treatment with G-TPP but not at 4 or 24 h, TBK1 was phosphorylated. (**B**) HeLa cells stably expressing EGFP-Parkin were treated with 10 µM G-TPP and fixed 8 h after treatment. Cells were stained with antibodies against the autophagy adapter proteins NBR1, NDP52, OPTN, p62, and TAX1BP1 (red). Mitochondria were counterstained with TOM20 antibodies (cyan), nuclei with Hoechst (blue). EGFP-Parkin epifluorescence is shown in green. Scale bar corresponds to 10 µM. (**C**) HeLa cells stably expressing EGFP-Parkin and the reporter protein mitoKeima were treated with 10 µM CCCP or G-TPP and imaged over time. The ratio of ‘neutral’ mitoKeima to ‘acidic’ mitoKeima was calculated as readout for mitophagy. Parkin translocation was monitored at the same time. Values for Parkin translocation and mitophagy were normalized to 12 h treatment with 10 µM CCCP as positive control and DMSO as negative control (two-way ANOVA with Tukey’s post-hoc test, ^**^p < 0.005, ^***^p < 0.0005).

To test induction of autophagy in general, and mitophagy in particular, we analyzed different marker proteins on western blot and by immunofluorescence. Treatment of HeLa cells with G-TPP increased levels of microtubule associated protein light chain 3B (LC3), both the processed form LC3-I and the lipidated form LC3-II. In addition, we observed phosphorylation of tank-binding kinase 1 (TBK1) in response to G-TPP by western blot (Figure 3A). TBK1 is activated by phosphorylation of Ser172 in its activation loop in a PINK1- and Parkin-dependent manner [23]. Downstream of this, at least five autophagy receptors have been implicated in mitophagy: NBR1, NDP52, p62/SQSTM1, OPTN and TAX1BP1. These bind to Ub on the mitochondrial surface and also bind to LC3 and other GABARAP family members to tether mitochondrial cargo to the forming autophagosome. Phosphorylation of these adaptors by activated TBK1 increases their recruitment to mitochondria [23]. Similar to CCCP-induced depolarization of mitochondria, all five autophagy adaptors were recruited to mitochondria in response to G-TPP (Figure 3B).

To directly assess mitophagy flux in HeLa cells, we used mitoKeima, a mitochondrial targeted fluorophore that has different excitation spectra depending on pH [24]. In order to quantify mitophagy rates, we used a stable HeLa clone expressing mitoKeima in addition to EGFP-Parkin and calculated the ratio of ‘acidic’ to ‘neutral’ mitoKeima fluorescence [25]. Consistent with previous results, the induction of Parkin translocation upon G-TPP was slower compared to CCCP (Figure 3C), although overall the curves for CCCP and G-TPP did not significantly differ ( *p* = 0.9607). Parkin translocation levels were decreasing 8 h after CCCP treatment, while G-TPP treated cells further increased Parkin translocation levels until 12 h of treatment. We observed a steady increase of mitophagy with CCCP over the 12 h time course in the same cells. Compared to DMSO treated cells G-TPP significantly induced mitophagy ( *p* < 0.0001), although less efficiently than CCCP ( *p* < 0.0001). Upon G-TPP, mitophagy peaked at 8 h and after 12 h the cells seemed to recover in line with what we observed for Parkin translocation and substrate degradation.

### G-TPP but not 17-AAG induces the PINK1/Parkin pathway

**Figure 4:**
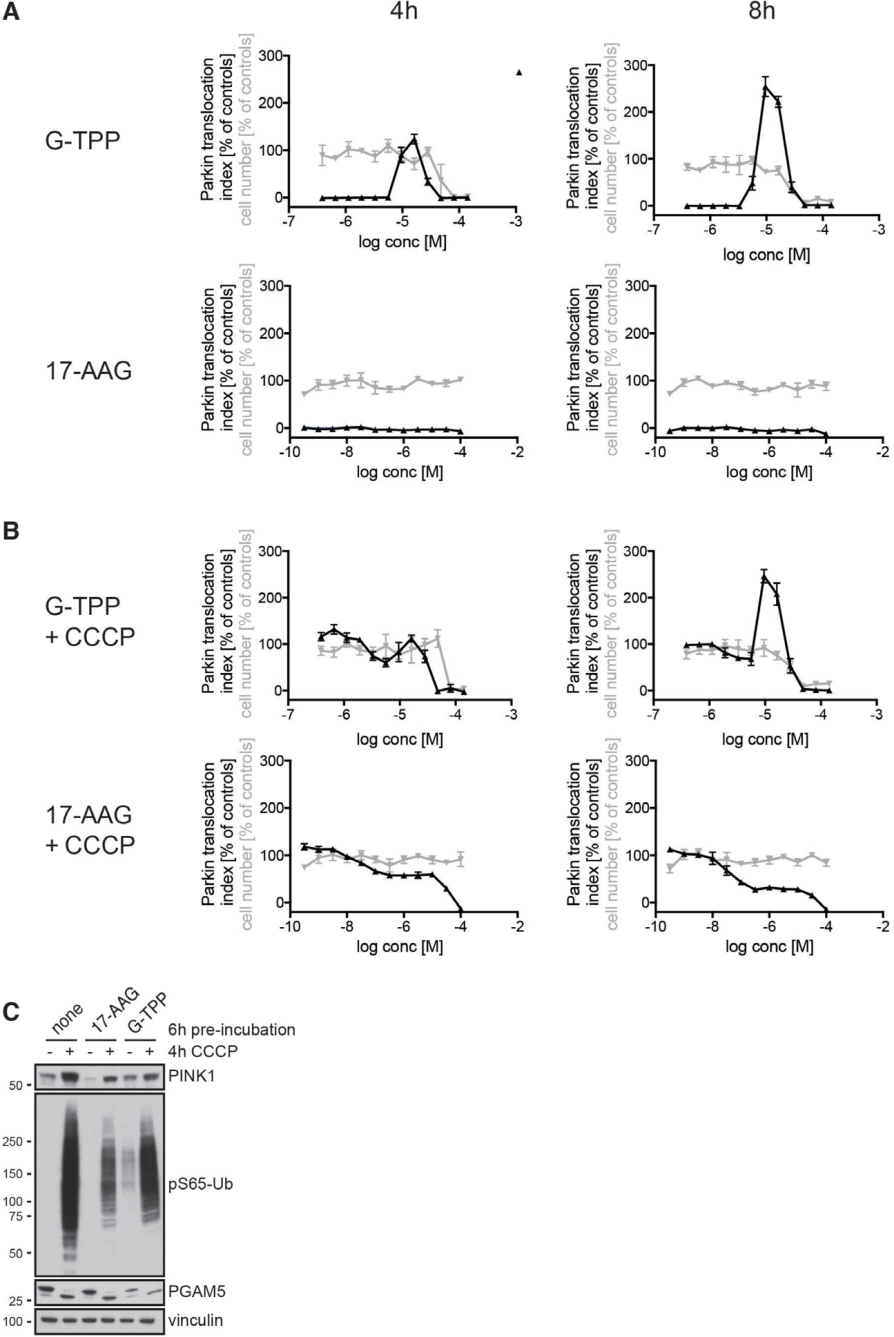
G-TPP but not 17-AAG induces Parkin translocation (**A**, **B**) HeLa cells stably expressing EGFP-Parkin were treated with 12 different concentrations of G-TPP or 17-AAG in a dose-response format for 2 or 6 h before adding (A) DMSO or (B) CCCP (final assay concentration = 10 µM) for an additional 2 h. Cells were fixed and analyzed for Parkin translocation (black line). Cell number was assessed by counting the number of Hoechst-positive nuclei in each well (gray line). Values were normalized to positive (2 h 10 µM CCCP) and negative (2 h DMSO) controls. In absence of CCCP, only G-TPP but not 17-AAG induced Parkin translocation. In combination with CCCP, increasing doses of 17-AAG led to inhibition of Parkin translocation. Very high concentrations of G-TPP also inhibited Parkin translocation and resulted in cell toxicity. (**C**) HeLa cells expressing untagged Parkin were treated with 1 µM 17-AAG, 10 µM G-TPP or DMSO as a control for 6 h before CCCP (10 µM) or medium containing DMSO was added for 4 h. Cells were harvested and western blots probed with antibodies against PINK1, pS65-Ub, the mitochondrial phosphatase PGAM5. Vinculin was used as a loading control. Compared to controls, 17-AAG pre-treated cells showed lower PINK1 levels that were accompanied by reduced pS65-Ub induction upon CCCP treatment, while G-TPP pre-treatment led to induction of pS65-Ub in the absence of CCCP, as expected.

We next used HCI to characterize the response upon treatment with G-TPP in detail and to test whether the cytosolic HSP90 inhibitor 17-AAG, which lacks the mitochondrial targeting moiety TPP, would also induce Parkin translocation (Figure 4A). HeLa EGFP-Parkin cells were treated with different amounts of either G-TPP or 17-AAG in dose response format and the results were normalized to 2 h 10 µM CCCP. In order to monitor cell death we assessed the cell number in each well by counting Hoechst-positive nuclei. In this setup, maximal Parkin translocation was obtained with 10–25 µM G-TPP. While these concentrations had no effect on the cell number 4 h after treatment, higher concentrations (>50 µM) were toxic and showed reduced Parkin translocation. Cytosolic HSP90 inhibition with 17-AAG did not induce Parkin translocation at neither of the tested concentrations. Likewise, we could not observe an effect on cell number suggesting that both the ability to induce Parkin translocation and toxicity of the HSP90 inhibitor are specific for the mitochondria-targeted variant. In an attempt to assess whether the effects of HSP90 inhibition and CCCP would be synergistic, we pre-incubated the cells with different amounts of G-TPP or 17-AAG and added CCCP for the last two hours (Figure 4B). Combination with CCCP resulted in 100% Parkin translocation at low G-TPP concentrations, but did not result in a change of the G-TPP peak (Figure 4A and 4B), suggesting that G-TPP and CCCP act via different molecular mechanisms.Interestingly, 17-AAG dose-dependently (>30 nM) suppressed CCCP-induced Parkin translocation. It has been described previously that PINK1 binds to HSP90 and its co-chaperone CDC37 and that its stabilization is dependent on active HSP90 [26] and we confirmed this finding. 17-AAG pretreatment decreased PINK1 levels at steady-state and upon CCCP, which was accompanied by reduced induction of pS65-Ub, while levels and molecular size of PGAM5, a mitochondrial phosphatase that is CCCP-dependently cleaved, were not changed by 17-AAG (Figure 4C). Consistent with previous results, G-TPP treatment by itself (i.e. in the absence of CCCP) induced PINK1 and pS65-Ub levels. Compared to CCCP only however, G-TPP pretreated cells showed less PINK1 and pS65-Ub levels. Reduced PGAM5 levels suggest that this might be the result of considerable mitochondrial degradation.

### G-TPP induces mitochondrial stress different than CCCP

**Figure 5:**
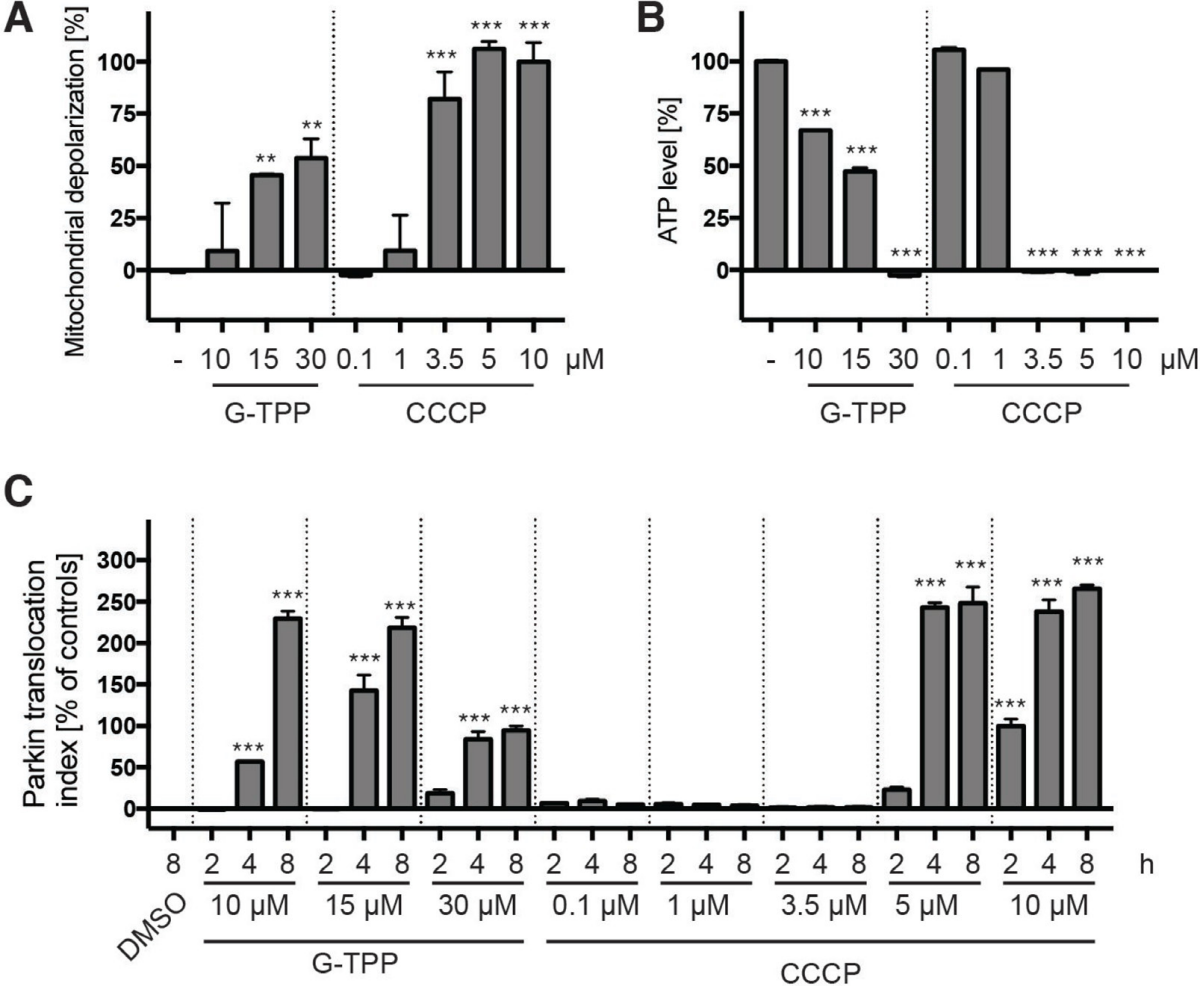
G-TPP induces mitochondrial stress different than CCCP (**A**) HeLa cells were treated with different amounts of G-TPP or CCCP for 4 h and loaded with the JC-10 dye. Green and red fluorescence was measured in a plate reader and expressed as a ratio normalized to negative (DMSO) and positive (10 µM CCCP) control wells. (**B**) Cells were treated in glucose-free medium as in (A) and ATP levels were analyzed with a luciferase assay. Values were normalized to negative (DMSO) and positive (10 µM CCCP) control wells. (**C**) HeLa cells stably expressing EGFP-Parkin were treated with the indicated concentration of G-TPP or CCCP for 2, 4 or 8 h. Control cells were treated with DMSO for 8 h. Cells were fixed and analyzed by HCI. Values were normalized to the negative control (8 h DMSO) and the positive control (2 h 10 µM CCCP). Shown is the mean ± SEM for 6 wells per treatment. Statistical analyses were performed with one-way ANOVA with Tukey’s post-hoc test, ^**^p < 0.005, ^***^p < 0.0005.

Given the different kinetics of CCCP and G-TPP, we decided to do a series of experiments to investigate the effects of G-TPP on mitochondria. First, we tested the consequences of G-TPP on mitochondrial membrane potential. HeLa cells were treated for 4 h with different concentrations of G-TPP, as indicated, loaded with JC-10 dye and analyzed with a plate reader (Figure 5A). Different concentrations of CCCP were used in parallel for comparison. In contrast to 10 µM CCCP, the concentration that is typically used to induce mitophagy in cell culture experiments, 10 µM G-TPP only led to partial mitochondrial depolarization, comparable to 1 µM CCCP. Even at 30 µM G-TPP the mitochondria were not fully depolarized. When we next measured ATP levels of cells treated the same way, we observed drastic depletion of cellular ATP with 30 µM G-TPP but not with 10 µM G-TPP (Figure 5B). All CCCP concentrations >1 µM led to a complete collapse of cellular ATP. When comparing the effect of G-TPP and CCCP on Parkin translocation side-by-side, we noticed that even though 3.5 µM CCCP had a stronger effect on the mitochondrial membrane potential and on cellular ATP levels than any of the G-TPP concentrations tested, 3.5 µM CCCP did not induce Parkin translocation, while G-TPP did (Figure 5C). We therefore concluded that G-TPP mediates the activation of PINK1 and Parkin at least partly through distinct mechanisms.

### G-TPP leads to induction of the mitochondrial unfolded protein response

**Figure 6:**
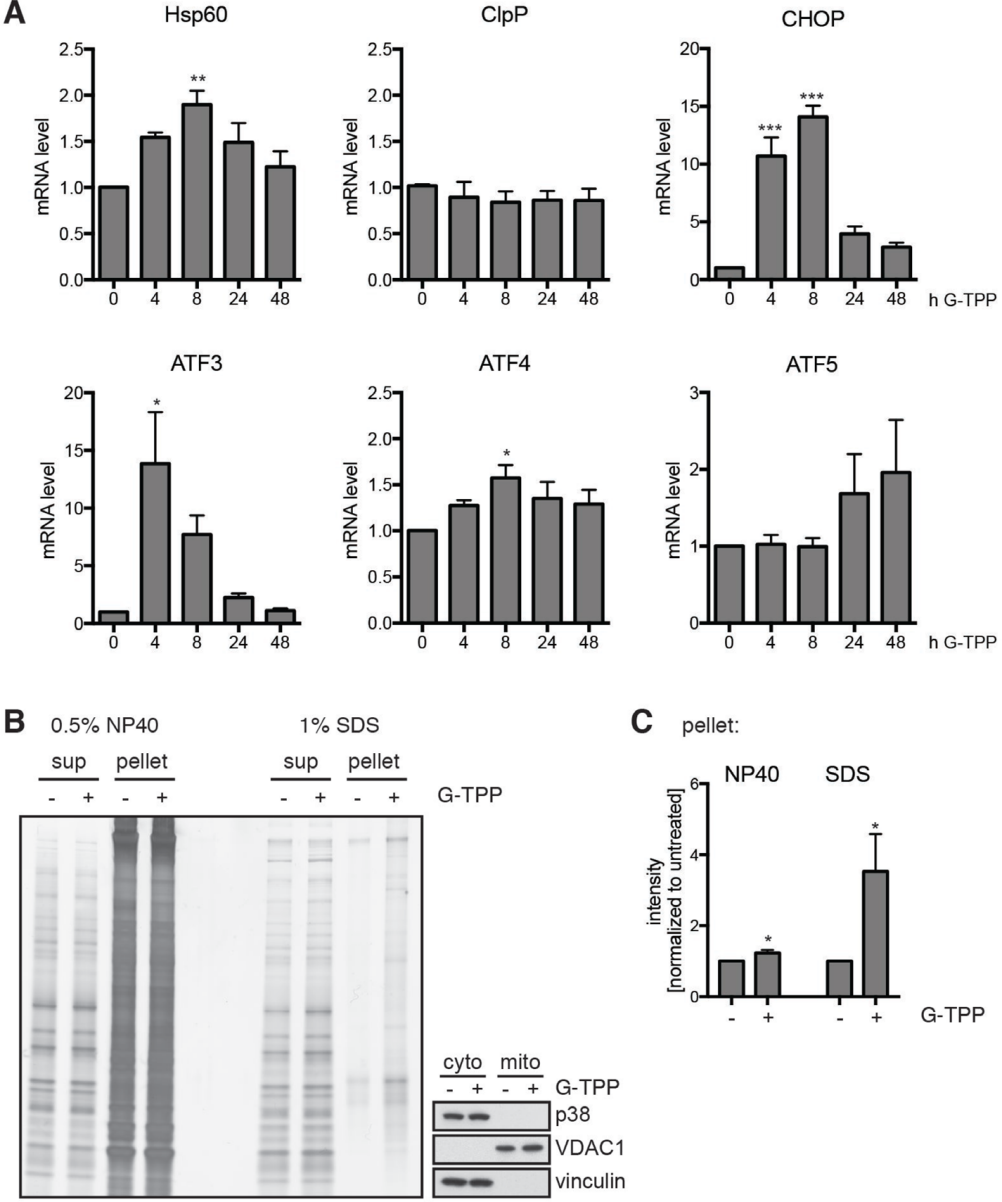
G-TPP induces the mitochondrial unfolded stress response (**A**) Quantitative RT-PCR with primer pairs specific to HSP60 (HSPD1) and ClpP (CLPP), CHOP (DDIT3), ATF3, ATF4, and ATF5. RNA was extracted from HeLa cells treated with G-TPP for the indicated times. Values were normalized to the housekeeping gene RPL27. Shown is the mean value of five independent experiments ± SEM (one-way ANOVA with Tukey’s post-hoc test, ^*^p < 0.05, ^**^p < 0.005, ^***^p < 0.0005). Cells treated with G-TPP showed induction of some but not all markers of the canonical mitochondrial unfolded protein response. ATF3 and ATF4, which have previously linked to the interconnected mammalian integrated stress response were significantly induced upon G-TPP. A trend for late induction of mitoUPR-associated ATF5 was also observed. (**B**, **C**) G-TPP treatment increased the amount of insoluble proteins in the mitochondrial fraction. HeLa cells were treated with G-TPP for 6 h. Cells were harvested and mitochondrial fractions prepared. NP40 and SDS soluble and insoluble proteins were subjected to SDS-PAGE followed by silver staining. The purity of mitochondrial and cytosolic fractions was verified using anti-p38 and vinculin as cytoplasmic and VDAC1 as mitochondrial marker. (C) Shown is the average of five independent experiments (one-way ANOVA with Tukey’s post-hoc test, ^*^p < 0.05).

Given that G-TPP treatment interferes with protein folding inside mitochondria, we tested the activation of mitochondrial unfolded protein (mitoUPR) response characterized by induction of genes encoding proteases and chaperones [27]. On RNA level we observed that HSP60, but not ClpP, was significantly induced (Figure 6A). In addition, G-TPP significantly induced the activating transcription factors ATF3 and ATF4 as well as the ATF4/ATF5 downstream target CHOP that have been associated with mitoUPR and the interconnected mammalian integrated stress response [20, 28]. A trend to later induction of ATF5, a mitoUPR gene that has been linked to apoptosis in response to protein homeostasis [29, 30] was also observed. To test if G-TPP indeed leads to the accumulation of unfolded proteins in mitochondria, we used mitochondrial preparations for fractionation into soluble and insoluble proteins with NP40 or SDS as detergents. Silver staining was used to detect proteins (Figure 6B). We observed an increase of NP40 and SDS insoluble protein in mitochondria from cells treated with G-TPP compared to untreated cells (Figure 6C). This suggests that G-TPP induces the accumulation of unfolded mitochondrial proteins in HeLa cells consistent with previous findings [19, 20].

### G-TPP induces mitophagy in primary human fibroblasts and iNeurons

**Figure 7:**
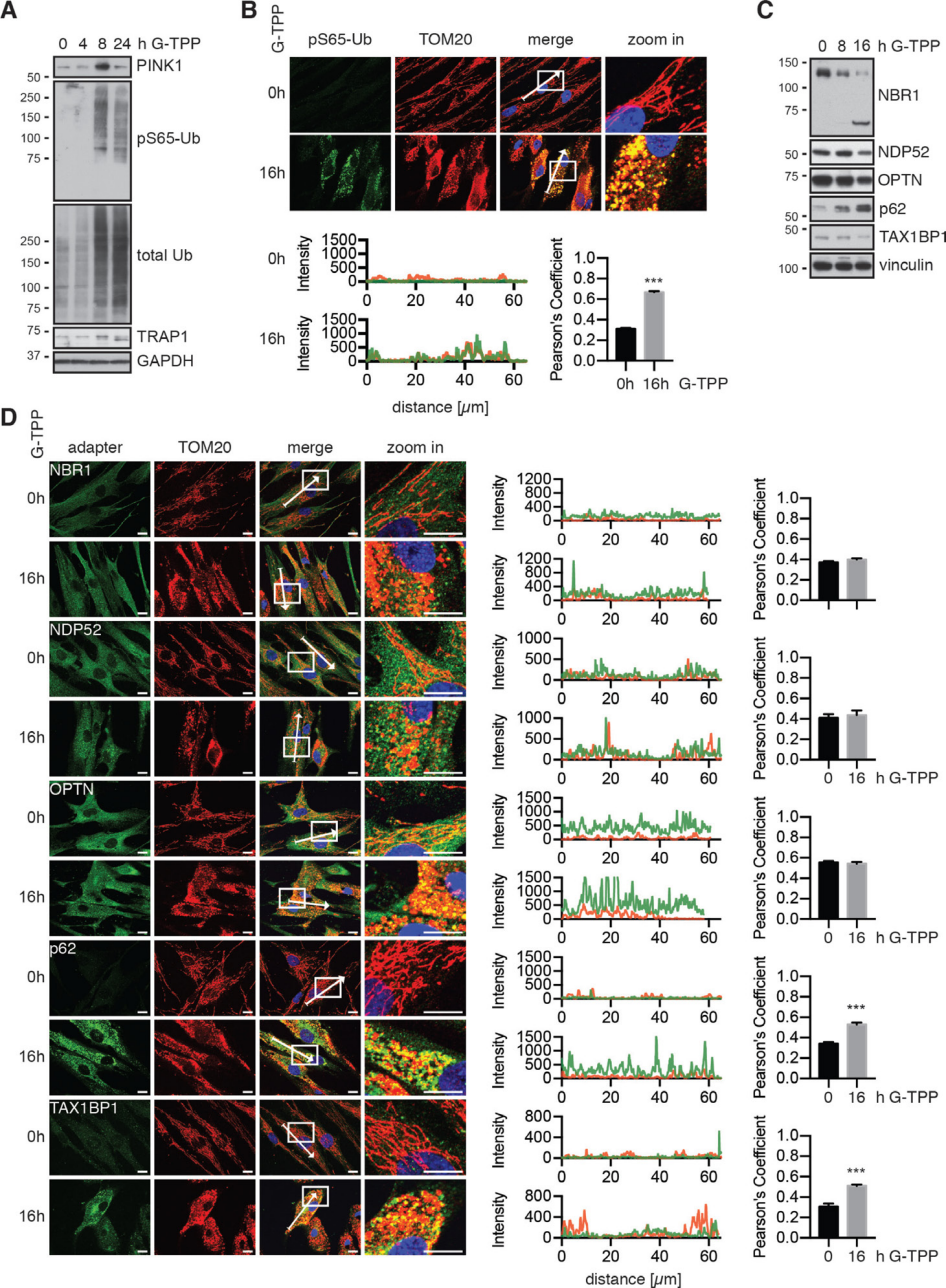
G-TPP activity is conserved in primary fibroblasts (**A**, **C**) Fibroblasts were treated with 15 µM G-TPP for the indicated time points. Cells were harvested and western blots were probed with antibodies against (A) PINK1, pS65-Ub and total Ub or (C) autophagy adapter proteins. GAPDH and Vinculin served as loading control. G-TPP treatment led to PINK1 stabilization and pS65-Ub induction in primary skin fibroblasts. p62 levels were induced upon G-TPP treatment, while other adapters seemed decreased. (**B**, **D**) Human fibroblasts were treated with 15 µM G-TPP for 16 h and fixed and stained with antibodies against (B) pS65-Ub (green) or (D) the autophagy adapters NBR1, NDP52, p62, OPTN and TAX1BP1 (green). Mitochondria were stained with antibodies against TOM20 (red), nuclei were visualized with Hoechst (blue). Scale bars indicate 10 µM. A magnified image of the boxed region, the fluorescence profile along the arrow and the Pearson’s correlation coefficient of adapter protein and mitochondrial stainingare shown to the right. Shown is the mean ± SEM of at least five randomly selected images (unpaired, two-sided *t*-test, ^***^p < 0.0005).

Though the effects of G-TPP in tumor / cancer cells have been analyzed in detail [19], not much is known about the consequences of G-TPP in primary cells. Using western blot and immunofluorescence staining of primary human fibroblast cells, we found stabilization of PINK1 protein 8 h and 16 h after treatment and robust induction of pS65-Ub (Figure 7A, 7B and Supplementary Figure 1A). Upon treatment with G-TPP, mitochondrial morphology changed and they appeared fragmented, similar to cells treated with CCCP or valinomycin (Figure 7B, Supplementary Figure 1A). This was independent of PINK1 and occurred in cells with a homozygous PINK1 loss-of-function mutation [31] (Supplementary Figure 1B). We monitored the levels and the recruitment of all five autophagy receptors in human fibroblasts (Figure 7C, 7D). Full-length forms of NBR1, NDP52 and OPTN all seemed decreased upon G-TPP, while p62 was robustly induced. For NBR1 we observed a smaller molecular weight band that increased at the same time the full-length band disappeared. On the single cell level, we detected speckle formation upon G-TPP treatment for all adapters (Figure 7D), but observed the most notable re-localization and partial co-localization with mitochondria for p62 ( *p* < 0.0001) and TAX1BP1 ( *p* = 0.0002), suggesting that G-TPP also induces mitophagy in primary cells.

**Figure 8:**
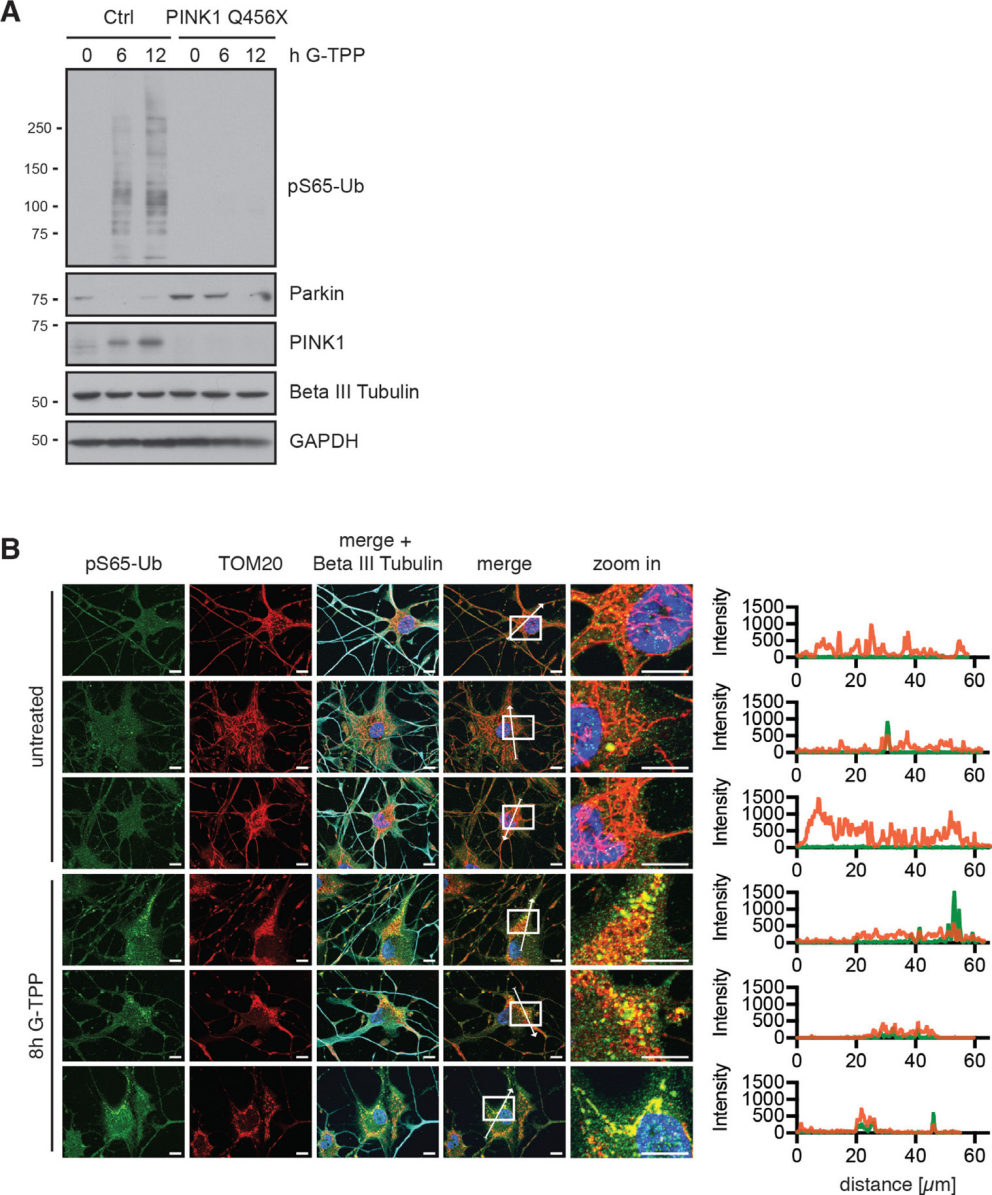
G-TPP triggers PINK1 activation in iNeurons (**A**) Control cells or PINK1 Q456X fibroblasts were converted to induced neurons (iNeurons). Cells were treated for the indicated times with 10 µM G-TPP and harvested. Western blots were prepared and probed with antibodies against PINK1, pS65-Ub and Parkin. Beta III tubulin served as a control for successful conversion to neuronal cells, GAPDH as a loading control. (**B**) iNeurons were treated with 15 µM G-TPP for 8 h and fixed. Cells were stained with antibodies against pS65-Ub (green) and the mitochondrial marker TOM20 (red) and the neuronal marker Beta III tubulin (cyan). Nuclei were stained with Hoechst 33342 (blue). Scale bars correspond to 10 µM. A magnified image of the boxed region and the fluorescence profile along the arrow are shown to the right.

Consistent with the effects on fibroblasts, we observed PINK1 stabilization and induction of pS65-Ub in neuron cultures obtained by directly converting skin fibroblasts (Figure 8A, 8B). Using Parkin antibodies that detect the unmodified/inactive conformation of Parkin, we observed that Parkin disappeared upon treatment with G-TPP in cells with functional PINK1, indicating its activation. In addition to iNeurons, we also treated primary mouse neurons with G-TPP. While they tolerated other mitochondrial stressors such as antimycin A (4 µM) /oligomycin (10 µM) or valinomycin (1 µM) quite well, they were very sensitive towards G-TPP and died already at very low concentrations, precluding analysis of PINK1 protein levels or kinase activity (data not shown).

## DISCUSSION

While the disease-relevant molecular mechanisms that induce mitoQC remain unclear, here we show that chemical inhibition of mitochondrial HSP90s by G-TPP treatment leads to activation of PINK1 and Parkin and downstream mitophagy in human cells. At the used sublethal dose, G-TPP is known to induce mitoUPR [20] and autophagy [19]. In addition, the activation of PINK1/Parkin-dependent mitophagy during mitoUPR has been described before [18]. However, in these instances mitoUPR was induced genetically by expression of the unfolded matrix protein ∆OTC or by knockdown of mitochondrial HSP70 [18, 32]. Consistently, G-TPP allows the pharmacological induction of PINK1 and Parkin-dependent mitophagy in cellular models independent of mitochondrial membrane depolarization or genetic manipulations.

G-TPP was developed to kill tumor cells. Mitochondria play a critical role in the neoplastic transformation of tumor cells as they regulate and maintain key metabolic and cell survival routes [33, 34]. Inhibition of mitochondrial HSP90 proteins provides an attractive strategy to combat cancer. First, since their levels are elevated in cancer the therapy targets specifically the tumor and second they are critically involved in cell survival pathways. Increased expression of TRAP1 is linked to multiple mitochondrial adaptations of cancer cells [34]. TRAP1 suppresses oxidative stress and prevents opening of the mitochondrial permeability transition pore [35]. Interestingly, TRAP1 is directly phosphorylated by PINK1 [36] and overexpression of TRAP1 rescues PINK1, but not Parkin loss of function in Drosophila [37, 38] suggesting a role for TRAP1 downstream of PINK1, but parallel to or upstream of Parkin. Knockdown of TRAP1 leads to a variety of mitochondrial related phenotypes that can be rescued by Parkin expression [37]. In HeLa cells knockdown of TRAP1 also induces HSP60 mRNA levels [20], but Parkin translocation to mitochondria has not been observed [4]. This suggests that, at least in this cell type, additional effects (e.g. mild depolarization, inhibition of mitochondrial resident HSP90s or increased oxidative stress) of G-TPP might be needed to induce PINK1/Parkin-dependent mitophagy. In contrast to TRAP1, induction of mitoUPR and increased mitophagy have been reported upon knockdown of the mitochondrial HSP70 family chaperone mortalin [32]. While reduced mortalin led to increased vulnerability toward apoptotic cell death, Parkin and PINK1 overexpression were able to mitigate the effects by increased lysosomal clearance [32].

Compared to classical inducers of mitophagy that disrupt the mitochondrial membrane potential (CCCP, valinomycin, antimycin/oligomycin), PINK1 accumulation, Parkin activation and recruitment as well as induction of mitophagy were slower. This is in line with the proposed mechanism of G-TPP. While the primary effect, i.e. the inhibition of chaperones, occurs immediately the downstream stress response will only be initiated after unfolded proteins begin to accumulate. Consistent with the induction of mitoUPR by G-TPP, we found elevated levels of insoluble proteins in mitochondrial fractions. While we also observed secondary loss of membrane potential and ATP depletion in G-TPP treated HeLa cells to a certain extent, these effects were not sufficient to explain activation of PINK1 and Parkin. Side by side comparisons with CCCP revealed that a low CCCP concentration that led to stronger effects on membrane depolarization and ATP levels was not able to trigger Parkin translocation to mitochondria. Accumulation of unfolded proteins inside mitochondria might lead to impaired protein import [18]. As a result, PINK1, which is imported into mitochondria and cleaved by mitochondrial proteases, is rerouted to the OMM [18]. Accumulation of PINK1 on the OMM can also be triggered by knockdown of the mitochondrial protease Lon [18, 39, 40] and is sufficient to induce Parkin activity and downstream mitophagy [6, 8].

While G-TPP induces PINK1 stabilization, induction of pS65-Ub and Parkin recruitment at earlier time points, the effects on cells upon longer treatment were less pronounced compared to CCCP. G-TPP significantly induced mitophagy but compared to CCCP the proportion of mitochondria undergoing mitophagy was much smaller. Indeed after prolonged incubation with G-TPP the cells seemed to recover from stress and 24 h after treatment many cells resembled untreated cells in terms of Parkin localization and mitochondrial morphology in immunofluorescence experiments. This was consistent with western blot analyses where after initial degradation the levels of several mitochondrial proteins normalized. It is known that mitoUPR triggers a general cellular response to alleviate this stress [41]. It remains unclear how these two pathways are connected and regulated and what factors decide whether to repair or degrade the mitochondria that harbor unfolded proteins.

In HeLa cells, we confirmed that the activity of G-TPP is caused by targeting specifically the mitochondrial pool of HSP90 as non-mitochondrial targeted version 17-AAG did not induce PINK1/Parkin. Interestingly, using 17-AAG we observed inhibition of Parkin translocation upon CCCP treatment at higher concentration. This is consistent with a role of cytosolic HSP70 and HSP90 for PINK1 import into mitochondria. Several PINK1 mutants have been shown to reduce binding to HSP90 [42]. This results in decreased stabilization of PINK1 [26]. In fact, western blot analysis revealed that PINK1 levels were reduced upon 17-AAG pretreatment. However, inhibition of cytosolic HSP90 could also have effects on other client proteins important for the PINK1/Parkin pathway. Altogether this points to an important role of HSP90 proteins for the induction of mitoQC pathways and the maintenance of mitochondrial health.

In mouse cells, mitochondrial HSP90 and TRAP1 are expressed in tumor cells and in high levels also in testis and brain [35]. In human cells, expression in normal cells can be observed although at much lower levels compared to cancer cells [35]. Consistently, we found G-TPP mediated activation of PINK1/Parkin not only in cancer cells (HeLa) but also in primary skin fibroblasts and thereof converted iNeurons. In line with high expression levels in mouse brain we found that G-TPP affected primary mouse neurons very strongly as they died at particularly low concentrations (5 µM). Expression of the unfolded matrix protein ΔOTC under the TH promoter led to neurodegeneration and motor behaviour impairment in mice [43]. This parkinsonian phenotype was further aggravated in a PINK1 KO background [43]. While the putative different roles that mitochondrial HSP90 chaperones and mitoUPR play in cancer and neurodegeneration remain unclear, it is interesting to note also PINK1 and Parkin have been suggested to play roles in both diseases [44].

## CONCLUSIONS

PINK1 and Parkin together mediate cytoprotective mitoQC. In cell culture models, mitophagy is routinely induced by treatment with mitochondrial uncouplers. However, the use of depolarizers has been criticized since uncoupling is not a physiologic trigger. In this study, we examined the cellular response towards G-TPP, a mitochondrial HSP90 inhibitor used for cancer treatment. In cancer but also primary cells, sub-lethal doses of G-TPP activate all facets of PINK1- and Parkin-dependent mitoQC. We conclude that G-TPP may represent a useful tool to study mitophagy via mitoUPR induction and independent from mitochondrial uncoupling.

## MATERIALS AND METHODS

### Cell culture

All cells were maintained at 37°C and 5% CO_2_ under humidified conditions. HeLa cells were obtained from ATCC. Control primary fibroblasts were from Cell Applications, Inc. PINK1 Q456X fibroblasts have been described before [31, 45]. HeLa cells and fibroblasts were cultured in Dulbecco’s modified eagle medium (DMEM, Invitrogen) containing 10% FBS (BioWest). Fibroblast medium was supplemented with 1% non-essential amino acid and 1% Penicillin/Streptomycin (both Invitrogen). HeLa cells stably expressing EGFP-Parkin, untagged Parkin, 3xFLAG-Parkin C431S or the EGFP-Parkin and mitoKeima have been described before [16, 21, 25]. iNeurons were generated as described [16].

### Antibodies

The following antibodies have been used for western blot (WB) or immunofluorescence (IF): beta III tubulin (#5568, CST, WB: 1/2,000 or AB9354, Millipore, IF: 1/250), FLAG (F3165, Sigma, WB: 1/150,000), GAPDH (H86504M, Meridian Life Sciences, WB: 1/500,000), LC3B (NB100-2220, Novus Biologicals, WB: 1/5,000), Miro1 (H00055288-M01, Novus Biologicals, WB: 1/500), Mitofusin 1 (ab57602, Abcam, WB: 1/5,000), Mitofusin 2 (ab56889, Abcam, WB: 1/5,000), NBR1 (H00004077-M01, Abnova, WB: 1/500, IF: 1/100), NDP52 (12229-1-AP, PTG, WB: 1/1,000, IF: 1/400), OPTN (sc-166576, Santa Cruz, IF: 1/100), OPTN (10837-1-AP, PTG, WB: 1/5,000), p38 (#9212, CST, WB: 1/2,000), p62 (610832, BD Biosciences, WB: 1/2,000, IF:1/500), Parkin (#4211, CST, WB: 1/3,000), PGAM5 (ab12653, Abcam, WB: 1/5,000), PINK1 (#6946, CST, WB: 1/2,000, IF: 1/1,000), PINK1 (BC100-494, Novus Biologicals, WB: 1/2,000), TAX1BP1 (#5105, CST, WB: 1/2,000, IF: 1/400), TBK1 (#3504, CST, WB: 1/1,000), pS172-TBK1 (#5483, CST, WB: 1/1,000), TOM20 rabbit (11802-1-AP, PTG, IF: 1/2,000), TOM20 mouse (sc-17764, Santa Cruz, IF: 1/100), TOM70 (14528-1-AP, PTG, WB: 1/5,000), TRAP1 (#13405, CST, WB: 1/1,000), ubiquitin (#3933, CST, WB: 1/2,000), pS65-Ub (in-house [16, 46], WB: 1/15,000, IF: 1/250), VDAC1 (ab14734, Abcam, WB: 1/10,000), vinculin (V9131, Sigma, WB: 1/500,000).

### Cell lysates and western blot

For whole cell lysates cells were washed twice in cold PBS and lysed in RIPA buffer (50 mM Tris pH 8.0, 150 mM NaCl, 1% NP-40, 0.5% deoxycholate, 0.1% SDS) containing protease and phosphatase inhibitor cocktails (Complete and PhosStop, Roche Applied Science). Lysates were incubated on ice for 30 min and spun at 14000 rpm, 4°C, to remove insoluble proteins. Protein concentrations were determined with BCA (Pierce BCA Protein Assay kit, Thermo Scientific). SDS–PAGE was performed using 8–16% Tris Glycine gels (Invitrogen). Proteins were transferred onto PVDF membranes and detected using standard immunoblotting procedures. For fractionation into mitochondrial and cytoplasmic fraction cells were washed, scraped in fractionation buffer (10 mM Tris pH 7.4, 200 mM mannitol, 1 mM EDTA, 50 mM sucrose) containing protease and phosphatase inhibitor cocktails and homogenized with 10 strokes through a 27G needle. Lysates were spun at 800 g for 5 min at 4°C to remove nuclei and post-nuclear supernatant was spun at 8000 g for 20 min at 4°C to pellet mitochondria. After measuring the protein concentration by BCA, equal volumes of 1% NP-40 or 2% SDS in cell lysis buffer (50 mM Tris pH 7.4, 150 mM NaCl, 1 mM EDTA) were added to 50 µg protein. Samples were spun at 45,000 rpm for 60 min. Supernatants were mixed with 6x Laemmli buffer, while pellets were dissolved in a sample buffer containing 8M Urea and 4% SDS. Samples were loaded onto 8–16% Tris-Glycine gels and silver staining was performed according to the manufacturers instructions (Pierce Silver Stain kit, Thermo Scientific). For Ub charging, cells were washed 2 times in PBS and lysed in prewarmed (95°C) SDS lysis buffer (50 mM Tris pH 7.6, 150 mM NaCl, 1% SDS). Lysates were homogenized by 10 strokes through a 23G needle. To verify the band shift by oxyester formation, aliquots of lysates were treated with NaOH (final concentration: 100 mM) for 1 h at 37°C loading onto SDS gels.

### Immunofluorescence staining

Glass cover slips were coated with PDL (Sigma-Aldrich) for HeLa cells and fibroblasts while growth-factor reduced matrigel (Millipore, 1/1,000 in PBS) was used for iNeuron experiments. Cells were fixed with 4% (w/v) paraformaldehyde (PFA), permeabilized with 1% Triton-X-100, blocked with 10% goat serum and incubated with primary antibodies followed by Alexa-488, -568 or -647 conjugated secondary antibodies (1/1,000, Invitrogen). Nuclei were stained with Hoechst 33342 (1/5,000, Invitrogen). Tyramide signal amplification (T20922, Invitrogen) was used for PINK1 staining. Coverslips were mounted onto microscope slides using fluorescent mounting medium (Dako). High-resolution confocal fluorescent images were taken with an AxioObserver microscope equipped with an ApoTome Imaging System (Zeiss).

### High content imaging

Cells were seeded onto 96- (BD Falcon) or 384-well (Greiner BioOne) imaging plates. For quantification of Parkin translocation HeLa cells stably expressing EGFP-Parkin were fixed with 4% PFA and stained with Hoechst. For quantification of mitophagy, HeLa cells stably expressing EGFP-Parkin and mitoKeima were seeded in in phenol-red free DMEM medium (Invitrogen) and imaged live. One hour before treatment Hoechst 33342 was added in a final dilution of 1/10,000. Cells were imaged in an incubation chamber at 37°C and 5% CO_2_ directly after addition of G-TPP (0 h) and after 4, 8 and 12 h. Acquisition was performed with a 2×2 montage (no gaps) after laser autofocus. 440/10 nM and 548/20 nM excitation filter were used for neutral and acidic Keima, respectively. Emission was filtered through a 595 nM longpass dichroic filter. Raw images were processed using the build-in AttoVision V1.6 software. Regions of interest (ROIs) were defined as nucleus and cytoplasm using the build-in ‘RING - 2 outputs’ segmentation for the Hoechst channel after applying a shading algorithm. Parkin translocation was calculated as described. For mitophagy, the signal intensity of the acidic mitoKeima in the cytoplasm was divided by the intensity of the neutral mitoKeima. Values were normalized to negative (DMSO) and positive controls (CCCP). Per experiment, at least three wells per condition with 300 cells were analyzed.

### qRT-PCR

Total mRNA from was prepared using the RNeasy mini kit (Qiagen) according to the manufacturer’s protocol and then reverse-transcribed using a High Fidelity cDNA kit (Roche Applied Science). cDNA was amplified by real-time PCR on a LightCycler 480 system (Roche) at 61°C annealing temperature using 2x Universal SYBR green mix (Bio-Rad) and specific primer pairs for Hsp60 (HSPD1) (For: ATTGCCAATGCTCACCGTAAGCC, Rev: CTGCCACAACCTGAAGACCAAC), ClpP (For:CGTATCATGATCCACCAGCCCTC, Rev: CCATGGCGGACTCGATCACCTG), ATF3 (For: GGAGCCTGGAGCAAAATGATG, Rev: AGGGCGTCAGGTTAGCAAAA), ATF4 (For: CAGCAAGGAGGATGCCTTCT, Rev: CCAACAGGGCATCCAAGTC), ATF5 (For: TGGCGACCCTGGGGCTGGAG, Rev: GGGCTCCCCCAAGGACCTCA) CHOP (DDIT3) (For: AGCCAAAATCAGAGCTGGAA, Rev: TGGATCAGTCTGGAAAAGCA), and Rpl27 as housekeeping gene (For: GATCGCCAAGAGATCAAAGATAAAA, Rev:CTGAAGACATCCTTATTGACGACAGT).

### Mitochondrial membrane potential and ATP measurements

To measure the mitochondrial membrane potential (mitochondrial membrane potential kit MAK-159, Sigma) cells were seeded in 20 µl in black 384-well plates with clear bottom in phenol-red free medium and treated the next day by adding 5 µl of 5x concentrated G-TPP or CCCP solutions. After 4 h incubation 12.5 µl of JC-10 dye in buffer A (1/100) was added to the cells and incubated for 45 min before 12.5 µl of buffer B was added. Plates were read on a Spectramax M5 plate reader (Molecular Devices) with a bottom-red using dual fluorescence (green: excitation 485 nM, emission 538 nM, cut-off 515 nM, red: excitation 544 nM, emission 590 nM, cut-off 570 nM). Ratio of green/red was used as extent of mitochondrial depolarization. Values were normalized to negative (DMSO) and positive controls (CCCP). For ATP measurements (CellTiter Glo, Promega) cells were seeded with 20 µl per well in white 384-well plates with clear bottom in phenol-red free and glucose free DMEM containing 25 mM galactose. The next day, 5 µl of a 5x concentrated G-TPP or CCCP solution was added to the wells and plates were incubated for 4 h at 37°C before they were equilibrated to room temperature. 25 µl ATP detection reagent was added to each well and mixed before luminescence was measured using a Wallac Victor 3V 1420 multilabel counter (Perkin Elmer). Per experiment, at least three wells per condition were analyzed.

### Statistical analysis

Statistical analyses between two groups were performed by unpaired, two-tailed *t*-test, between 3 or more groups with one-way or two-way ANOVA and Tukey’s posthoc test (^***^p < 0.0005, ^**^p < 0.005, ^*^p < 0.05) using GraphPad Prism version 7.

## SUPPLEMENTARY MATERIALS FIGURE



## Abbreviations

17-AAG: 17- (Allylamino)-17-demethoxygeldanamy cin
ATP: adenosine 5′-triphosphate
CCCP: carbonyl cyanide *m*-chlorophenyl hydrazine
CHOP: C/EBP homologous protein
DMSO: dimethyl sulfoxide
EGFP: enhanced green fluorescent protein
G-TPP: Gamitrinib-triphenylphosphonium
HCI: high content imaging
HSP60: Heat shock protein of 60kDa
HSP70: Heat shock protein of 70kDa
HSP90: Heat shock protein of 90kDa
IF: immunofluorescence
IMM: inner mitochondrial membrane
LC3: microtubule associated protein light chain 3B
mitoKeima: mitochondrial targeted Keima reporter protein
mitoQC: mitochondrial quality control
mtDNA: mitochondrial DNA
MPP: matrix processing peptidase
MTS: mitochondrial targeting sequence
NBR1: neighbor Of BRCA1 Gene 1
NPD52: nuclear dot protein 52
OMM: outer mitochondrial membrane
OPTN: optineurin
OTC: ornithine transcarbamylase
PARL: Presenilin-associated rhomboid-like protein
PD: Parkinson’s disease
PINK1: PTEN-induced putative kinase 1
p-S65-Ub: PINK1 phosphorylated ubiquitin (Serine-65)
qRT-PCR: quantitative reverse transcriptase polymerase chain reaction
SDS: Sodium dodecyl sulfate
TAX1BP1: Tax1 binding protein 1
TBK1: Tank-binding kinase 1
TIM: translocase of the inner mitochondrial membrane
TOM: translocase of the outer mitochondrial membrane
TRAP1: TNF-receptor associated protein 1
Ub: ubiquitin
WB: Western blot
