# Supplementary material for: Mitochondrial targeted HSP90 inhibitor Gamitrinib-TPP (G-TPP) induces PINK1/Parkin-dependent mitophagy
Source: Oncotarget. 2017 Nov 6;8(63):106233–48. doi: 10.18632/oncotarget.22287 (PMC5739729; doi:10.18632/oncotarget.22287)
Supplement: Supplementary file 1 [file oncotarget-08-106233-s001.pdf]

Mitochondrial targeted HSP90 inhibitor Gamitrinib-TPP (G-TPP) induces PINK1/Parkin-dependent mitophagy

SUPPLEMENTARY MATERIALS

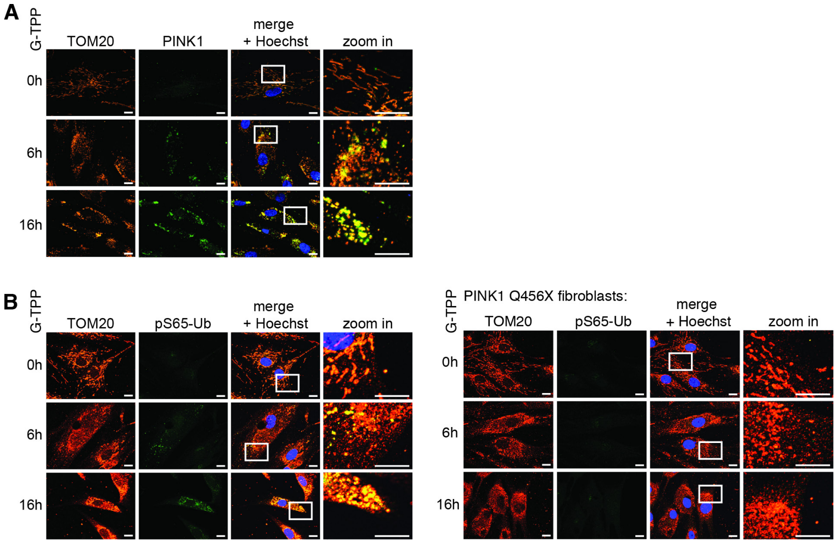

Supplementary Figure 1: (**A**, **B**) Fibroblasts were stained with antibodies against (A) PINK1 or (B) pS65-Ub (green). Mitochondria were stained with antibodies against TOM20 (red) and nuclei with Hoechst (blue). Scale bars correspond to 10 µm. The specificity of the pS65-Ub signal was confirmed by using a fibroblast line with a homozygous PINK1 Q456X loss of function mutation.
